# Der Gesellschaft naturforschender Freunde, &c.

**Published:** 1800-03

**Authors:** 


### NATURAL HISTORY.

*Der Gesellschaft naturforschender Freunde, &c.* — New Essays by the Society of Natural History at Berlin. Vol. II. with eight plates. Berlin. Printed for the Society, 1799.

The account, given in this volume, of the lives of those members of the Society, who have deceased before its publication, (amongst whom we find the names of Wildenman, Gren, Forster, and Hedwig) serve to render it peculiarly interesting. The Essays contained in it are the following:

I. Physico-mineralogical account of the gold and silver mines at Nazy Ag, in Transylvania; by Counsellor Stütz. This Essay is designed by its author, for a Supplement to Born's Mineralogical Letters upon Transylvania and Hungary. II. Account of some very rare plants, by C. L. Wildenow. III. Description of the Vittaria, a newly discovered genus of fern, by Olow Schwartz. In Smith's new classifications of ferns, we find the genus Vittaria; but he enumerates not more than a single species, the Pteris Liniata, of Linnæus. Mr. Schwartz describes two more species, the Vittaria lanceolata, and the Vittaria ensiformis, both of which are natives of the East Indies and the Island St. Maurice. IV. Observations respecting electrical motions, and the manner in which they are affected by pointed bodies; upon thunder, lightening, and conductors. A Dissertation read by Prof. de Luc to the Society. V. Mineralogical description of some fossils collected in the province of Sendomir, the greater part about Miedziara Gora, and in its vicinity, by Lieutenant General Baron L. von Geassau. VI.

Geological

Geological obfervations upon the preceding article, by M. Karften, Counfellor of Mines. VII. Inquiry into the fiery meteors obferved in the atmofphere, by Baron von Halin. VIII and IX. Of two copper ores found in Siberia, by J. J. Bindheim, Apothecary in Mofcow. X. Of the Chalcedony of Siberia and Dauria, by the fame. XI. Account of fome obfervations and experiments, by Baron von Gerfdorf. XII. Geological obfervations upon a part of the mountains of Schwartzwald, by J. F. Wildenman. XIII. Mineralogical defcription of the ftratum of pit-coal found in fome of the mountains of the Lingen territory, by Counfellor Karften. XIV. Of the rotatory ofcillations of a pendulous beam, by E. F. Chladni. XV. Account of fome fingularities in the iron ores of Stachenburg and Ifenburg, by Mr. Cramer. XVII. Thoughts refpecting the fuppofed alterations of the zones of the earth, by Prof. Bode. XVIII. Chemical experiments and obfervations, relating to the production of fugar, or fyrup, from indigenous vegetables of Germany, by S. F. Hermftärdt. According to thefe experiments, the different fpecies of the acer, (viz. the acer dafycarpum, Erharti, and faccharinum, of Linnæus) feem preferable to all other European plants, for affording a fubftitute for the fugar-cane. XIX. Extracts from letters.

